# Prevalence of Homologous Recombination Deficiency and Treatment Patterns in Patients with Newly Diagnosed Advanced Ovarian Cancer in Bulgaria: A Real-World Cohort Study (VALIDATE)

**DOI:** 10.3390/medicina62051000

**Published:** 2026-05-21

**Authors:** Jeliazko Arabadjiev, Krasimir Nikolov, Marchela Koleva, Nikolay Shopov, Ivan Tonev, Rossitza Krasteva, Ivan Donev, Valeriy Yordanov, Velko Minchev, Assia Konsoulova

**Affiliations:** 1 Medical Oncology Clinic, Acibadem City Clinic University Hospital Tokuda, 1407 Sofia, Bulgaria; jarabadjiev@gmail.com; 2 Medical Oncology Department, Complex Oncology Center–Burgas, 8000 Burgas, Bulgaria; kznikolov@abv.bg; 3 Medical Oncology Department, MHAT “Sveta Sofia”, 1618 Sofia, Bulgaria; m_d_koleva@abv.bg; 4 Medical Oncology Department, MHAT Central Onco Hospital, 4000 Plovdiv, Bulgaria; nikolay.at.shopov@gmail.com; 5 Medical Oncology Department, Complex Oncology Center–Plovdiv, 4002 Plovdiv, Bulgaria; dr.ivan.tonev@gmail.com; 6 Medical Oncology Department, Uni Hospital Oncology Center, MHAT Uni Hospital, 4500 Panagyurishte, Bulgaria; rkr_2002@yahoo.com; 7 Medical Oncology Department, MHAT Women’s Health Hospital Nadezhda, 1330 Sofia, Bulgaria; ivan_donev75@abv.bg; 8 Medical Oncology Department, Complex Oncology Center–Ruse, 7002 Ruse, Bulgaria; dr_vjordanov@abv.bg; 9 Medical Oncology Department, UMHAT Sofiamed, 1797 Sofia, Bulgaria; v_minchev@abv.bg; 10 Bulgarian Joint Cancer Network, Women for Oncology–Bulgaria, Medical Oncology Department, USHATO “Prof. Ivan Chernozemski”, 1756 Sofia, Bulgaria

**Keywords:** advanced ovarian cancer, homologous recombination deficiency, genomic testing, *BRCA*, genomic instability, Bulgaria

## Abstract

*Background and Objectives*: Although clinically useful, homologous recombination deficiency (HRD) testing has recently been more broadly adopted in ovarian cancer (OC) management. The VALIDATE study evaluated HRD status and treatment patterns in patients with newly diagnosed advanced OC in Bulgaria to better understand HRD prevalence and disease management. *Materials and Methods*: This real-world, observational, multi-centre, medical chart review study included 100 adult patients with HRD testing results available at study entry. Data collected at least 30 days after HRD results and 6 months later were descriptively analysed in the full cohort and subgroups (HRD, *BRCA* mutation, and genomic instability score [GIS]). *Results*: Mean age at diagnosis: 61.3 years; stage III: 51.0%, prevalence of HRD+ 58.0% (95% confidence intervals [CI] 47.7–67.8%) and HRD− 42.0% (95% CI 32.2–52.3%). Among the 58 HRD+ patients, 20 (34.5%) were *BRCA*+, whereas 38 (65.5%) were *BRCA*−, and 52 (89.7%) were GIS+, and 6 (10.3%) GIS−. Overall, platinum–taxane chemotherapy plus antiangiogenics was the most common front-line (FL) treatment (77.0%), regardless of subgroups (range: 66.7–85.0%). Six months later, 81 patients were alive, and 73 (90%) started maintenance therapy (MT). Antiangiogenic monotherapy (32.0%) and antiangiogenic plus PARP inhibitor (34.0%) were the most common MTs. The latter was also common across subgroups (range: 33.3–60.5%), except for HRD− (61.9% received antiangiogenic monotherapy). *Conclusions*: In this dataset, more than half of advanced OC patients had HRD+ status. Our study provides relevant insights into recent clinical practice patterns in advanced OC in Bulgaria that could serve as an anchor for future, more robust research in this field.

## 1. Introduction

Ovarian cancer (OC) remains the main cause of death among gynaecological malignancies, representing a significant proportion of cancer-related fatalities among women worldwide [1]. Globally, Europe reports the highest incidence of OC in 2022, with a rate of 18.0 per 100,000 women/year and the highest associated mortality rate of 12.0 per 100,000 women/year [2]. Most recent estimates indicate that from 2022 to 2050, both the incidence and mortality from OC will continue to rise, with the number of new cases increasing by approximately 9% and deaths by nearly 20% [3]. Bulgaria ranks among the top ten European countries in terms of OC incidence in 2022, with a crude incidence rate of 19.2 per 100,000 women/year and an associated mortality rate of 12.9/100,000 [2,4], emphasizing a high disease burden. Since this data mostly originates from external sources, tracking real-time cancer incidence, prevalence, and treatment outcomes specific to the Bulgarian population is challenging. This gap highlights the need for a national cancer registry to improve data collection, treatment planning, and health policy development.

Despite advancements in surgical cytoreductive techniques and maintenance (MT) therapy with platinum and taxanes, the 5-year survival rate for OC remains below 50% [5]. This is likely due to its nonspecific clinical symptoms, delayed diagnosis, advanced disease stage at diagnosis, chemoresistance, and disease recurrence [1,6,7]. Additionally, most patients with advanced OC have limited options for disease control after front-line (FL) systemic therapy due to various factors, including poor performance status, adverse events, tumour histology, duration of response to the initial treatment, and the tumour’s genetic profile. These factors may affect the feasibility and effectiveness of subsequent therapies [8]. While no public health screening programs are currently in place for women at average risk, selected high-risk individuals may benefit from surveillance programs in certain countries [9,10,11]. However, Bulgaria does not currently offer such programs for women at high risk.

OC is highly heritable and is associated with germline mutations, mainly in the *BReast CAncer* genes (*BRCA1* and *BRCA2*) [1,11]. Carriers of pathogenic *BRCA1* mutations have an overall risk of 18% to 54% of developing OC, while *BRCA2* pathogenic mutation carriers have a risk of 2.4% to 19% by age 70 years [12]. In advanced OC, approximately 50% of tumours have a homologous recombination deficiency (HRD), with 13–15% attributable to pathogenic germline *BRCA* mutations, 5–7% to somatic *BRCA* mutations, and nearly 30% to non-*BRCA* homologous recombination repair (HRR) gene alterations [13,14].

Current guidelines base treatment decisions in OC on HRD status, *BRCA* mutation status, and genomic instability score (GIS) [14]. In Bulgaria, local guidelines for OC management align with international treatment protocols. This is crucial in the era of targeted therapies, as HRD+ tumours are sensitive to poly (adenosine diphosphate [ADP]-ribose) polymerase (PARP) inhibitors, which have demonstrated efficacy regardless of *BRCA* mutation status [5]. Knowing HRD status is essential for an effective OC treatment strategy.

At the time of the present study, HRD testing was neither standardized nor implemented on a larger scale in Bulgaria, despite its utility for identifying patients with advanced OC sensitive to platinum salt chemotherapy and PARP inhibitors [9,15]. Currently, testing may still be underutilized due to reimbursement limitations. Given the instrumental role of this biomarker in individualizing the MT approach, it is important to understand the clinical profiles of patients with newly diagnosed OC in Bulgaria and treatment patterns.

The VALIDATE study collected recent real-world data on HRD status and treatment patterns in patients with newly diagnosed advanced OC, fallopian tube, or primary peritoneal cancer (hereafter referred to as advanced OC) in Bulgaria. We aimed to provide relevant insights into the decision-making parameters in everyday practice and to support the optimization of genetic testing for these patients.

## 2. Materials and Methods

### 2.1. Study Design and Population

VALIDATE was an observational, multi-centre, physician-led medical chart review study, conducted between May 2023 and July 2024 across 10 public or private medical centres across Bulgaria. Eligible participants were adult patients (aged ≥18 years) with a confirmed diagnosis of advanced OC who underwent HRD testing as part of routine clinical practice, had test results available at the study start, and provided written informed consent for study participation. Patients were considered ineligible if HRD status was tested as part of an interventional clinical trial, if they were part of a clinical trial at the time of enrolment, or if enrolment occurred <30 days after the HRD testing results became available. All patients eligible that were under the routine care of the study investigator at study start were invited to the informed consent discussion, and enrolled consecutively, in the order of their informed consent date.

Several timepoints were defined for this study. Index date was defined as the date on which HRD testing results became available, any time after 1 August 2022. Enrolment date was required to be at least 30 days after the index date, to maintain the non-interventional character of the study. The baseline period was defined as the interval between the OC diagnosis and index date (up to 6 months). The first round of data extraction was performed after enrolment, and the second round 6 months later, but ≥30 days after the patient’s last routine visit at the respective study site (Supplementary Figure S1).

The HRD testing was performed using the AmoyDx^®^ HRD Focus Panel (Amoy Diagnostics Ltd., Xiamen, Fujian, China), which is a next-generation sequencing (NGS) assay for qualitative detection of the deoxyribonucleic acid (DNA) abnormalities, where DNA is isolated from formalin-fixed paraffin-embedded tumour tissue specimens. It evaluates somatic *BRCA1/2* alterations and genomic scar score (GSS; GSS high: ≥50, GSS low: <50). Of note, in Bulgaria, the HRD testing is centralized and supported by the pharmaceutical industry. HRD testing is performed in two private laboratories that have been pre-qualified and externally assessed for NGS.

Study site physicians or delegated clinical research staff collected patient data from paper and/or electronic medical files. Data were collected in a single anonymized dataset using an electronic case report form (eCRF) accessible to the study site via a secure web link.

The study was performed in compliance with the ethical guidelines of the Declaration of Helsinki, and principles of Good Clinical Practice (GCP) and Good Pharmacoepidemiology Practice (GPP), including the archiving of essential documents. The National Ethics Committee approved the study conduct with approval number EKKИ/CT-0042/25 January 2023.

### 2.2. Study Objectives

The primary objective was to estimate the prevalence of HRD+ patients with newly diagnosed advanced OC, including the distribution of patients by *BRCA* mutation status and genomic instability, both as absolute numbers and relative frequencies.

The secondary objectives were to describe patient demographics, clinical characteristics, and treatment patterns in the overall cohort and in subgroups defined by HRD status, including *BRCA* mutation and genomic instability score (GIS) among HRD+ patients.

### 2.3. Study Outcomes

The following variables were collected at enrolment: patient characteristics (age, body mass index [BMI]), medical history (comorbidities of interest, personal history of breast cancer, family history of cancer), disease characteristics (primary tumour location, histological type, OC stage at diagnosis as assessed by The International Federation of Gynaecology and Obstetrics [FIGO] and TNM Classification of Malignant Tumours [TNM] staging criteria [16], Eastern Cooperative Oncology Group (ECOG) performance status (PS), cytoreductive surgery, serum cancer antigen-125 [CA-125] level), HRD testing results (including GSS, *BRCA* and GIS status), and FL treatment regimen.

At 6-month follow-up, disease characteristics (vital status, tumour response after FL chemotherapy, and serum CA-125 level) and MT characteristics (antiangiogenics, PARP inhibitors, combination of an antiangiogenic and a PARP inhibitor, other) were collected.

### 2.4. Statistical Analysis

As this was a real-world, observational study with primarily descriptive objectives, no formal hypothesis-based sample size calculation was performed. The target number of participants was determined based on feasibility and expected patient volume at participating sites. Considering the preliminary site feasibility assessments and the total annual number of patients newly diagnosed with advanced OC in Bulgaria [17], the planned sample size was 100 patients, with an initial quota of 10 patients per site. Based on the overall enrolment rate, larger sites were allowed to increase their quota.

Descriptive (pre-specified) analyses were performed on the overall cohort (full analysis set [FAS]) and subgroups stratified by HRD status (HRD+ and HRD−), presence of *BRCA1/2* mutations (*BRCA*+ and *BRCA*−), and GIS (GIS+ and GIS−).

Data were analysed using the R language (https://www-r-project.org/), version 4.2.2. Descriptive statistics included tabulation of frequencies, means with standard deviation (SD), medians with minimum (min) and maximum (max) for continuous variables, as well as frequencies and percentages for categorical variables. Two-sided, 95% confidence intervals (CIs) for the means and HRD-related key estimates were provided. The proportion of missing data was reported. Missing data were not imputed.

Exploratory statistical tests were performed to assess differences within groups of interest: student’s *t*-test (for continuous variables normally distributed) or Mann–Whitney–Wilcoxon (for ordinal variables). The difference was assessed as statistically significant if the *p*-value was <0.05. No adjustments for multiple comparisons have been applied.

## 3. Results

### 3.1. Prevalence of HRD+ Status in Patients with Advanced OC (Primary Objective)

In total, 100 patients newly diagnosed with advanced OC were included in the FAS. Of these, 58 (58.0% [95% CI 47.7–67.8%]) were HRD+ and 42 (42.0% [95% CI 32.2–52.3%]) were HRD−.

Among HRD+ patients, 20 (34.5% [95% CI 22.5–48.2%]) were *BRCA* mutation-positive (*BRCA*+ group) and 38 (65.5% [95% CI 51.9–77.5%]) were *BRCA*-negative (*BRCA*− group); whereas, 52 (89.7% [95% CI 78.8–96.1%]) were GIS-positive (GIS+ subgroup) and 6 (10.3% [95% CI 3.9–21.2%]) were GIS-negative (GIS− subgroup). In the *BRCA*+ group, 13 patients tested positive for *BRCA1* mutation, six for *BRCA2* mutation, and one for concurrent *BRCA1/2* mutations.

### 3.2. Patient and Disease Characteristics

In the FAS, the mean age (SD) at advanced OC diagnosis was 61.3 (10.8) years, with most patients (84.0%) aged over 50 years (Table 1). In the exploratory analyses of subgroups, no significant age difference was observed between HRD+ and HRD− groups (*p* = 0.82). In this HRD+ set, patients in the *BRCA*+ subgroup had a lower mean (SD) age versus those in *BRCA*− subgroup (56.1 [9.9] versus 63.8 [10.9] years, respectively, *p* = 0.0098; medium effect size *d_Cohen_* = 0.7256; Student’s test).

The most common comorbidity (46.0% of patients) was cardiovascular disease (CVD). Family history of malignancy was reported in only 19.0% of patients (Table 1). Stage III advanced OC was reported in 51% of patients, with no differences (Mann–Whitney–Wilcoxon test) between the subgroups stratified by HRD (*p* = 0.3), *BRCA* (*p* = 0.43) or GIS status (*p* = 0.31).

Most patients (98.0%) in this dataset had an ECOG PS of 0–1 at diagnosis (Table 1), with differences between the distribution of patients by ECOG PS in HRD− and HRD+ subgroups (ECOG PS 1: 52.4% versus 25.9%; ECOG PS 2: 4.8% versus 0.0%, respectively; *p* = 0.0012; medium effect size *δ_Cliff_* = 0.33; Mann–Whitney–Wilcoxon test). No other significant differences were observed between other subgroups.

Baseline serum CA-125 levels were available for 78 (78.0%) patients, of whom 75.6% had elevated levels (>35 U/mL). Subgroup details are presented in Supplementary Table S1a. Most patients (71.0%) underwent cytoreductive surgery (CS), of whom 57.0% had primary CS (Table 1). Negative margins were recorded in 45.1% (*n* = 32) of patients, positive microscopic margins of <1 cm in 7.0% (*n* = 5), macroscopic residual disease remaining ≥1 cm in 19.7% (*n* = 14), and the CS outcome was not available in 28.2% (*n* = 20). Similar trends for CS type and margins of surgical resection were observed in the subgroups (Table 1). The mean (SD) GSS was 50.4 (42.6), with higher values observed in the HRD+ group (81.4 [27.9]) versus HRD− group (7.7 [9.2]). GSS detailed results in subgroups are shown in Supplementary Table S2.

### 3.3. Front-Line (FL) Treatment Patterns

In the FAS, the mean (SD) time from advanced OC diagnosis to FL treatment initiation was 1.6 (1.3) months, with a median (min, max) of 1.4 months (0.1, 9.3). One HRD+, *BRCA*+, and GIS+ patient who started treatment before a confirmed advanced OC diagnosis (initially diagnosed with endometrial cancer) was excluded from this analysis. Across all subgroups, the median time from diagnosis to FL treatment initiation was similar, of approximately 1 month.

**Figure 1:**
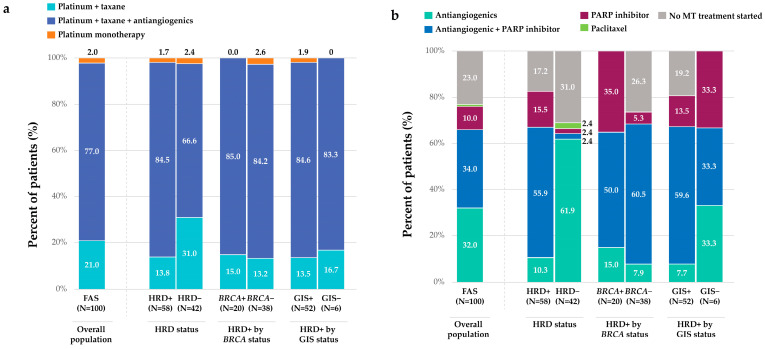
Real-world treatment patterns in patients newly diagnosed with advanced OC (FAS and by subgroups): (**a**) FL treatments, (**b**) MT treatment. Notes: 1. In the overall population (FAS), 13 patients were lost to follow-up: 2 died before MT treatment started, and among those who were alive, 8 did not start any MT treatment. 2. In the HRD− subgroup, 1 patient received antiangiogenic therapy + PARP inhibitor following the identification of a germinal *BRCA*+ mutation, and 1 patient received PARP inhibitor as the ultimate option (antiangiogenic therapy contraindicated). Abbreviations: *BRCA*(+/−) = *BReast Cancer* gene (positive/negative); FAS, full analysis set; FL = front-line; GIS(+/−) = genomic instability score (positive/negative); HRD(+/−) = homologous recombination deficiency (positive/negative); MT = maintenance; N = total number of patients; OC = ovarian cancer; PARP = poly ADP-ribose polymerase.

The most commonly used FL treatment was platinum–taxane doublet chemotherapy combined with antiangiogenic therapy in both FAS (77.0%) and subgroups of interest (range: 66.7–85.0%). Platinum–taxane doublet chemotherapy alone was used mainly in the HRD− group (Figure 1a). The exploratory analyses between subgroups showed no significant differences in the distribution of FL treatment.

At 6-month follow-up, 81.0% of patients (*n* = 81) included in the FAS were alive, 6.0% (*n* = 6) were deceased, and 13.0% (*n* = 13) were lost to routine follow-up. Among 81 survivors, 90.1% (*n* = 73) started MT, of whom 97.9% (*n* = 46 of 47) were in the HRD+ group and 79.4% (*n* = 27 of 34) in the HRD− group; 100% (*n* = 19 of 19) were in the *BRCA*+ subgroup and 96.4% (*n* = 27 of 28) in the *BRCA*− subgroup; and 97.5% (*n* = 40 of 41) were in the GIS+ subgroup and 100% (*n* = 6 of 6) in the GIS− subgroup.

Among non-survivors, 33.3% (*n* = 2) died before starting MT and 67.7% (*n* = 4) after its initiation. Among HRD+ deceased patients (*n* = 3), one was *BRCA*+, 2 were *BRCA*−, and all were GIS+. The mortality rate was similar between the *BRCA*+ and *BRCA*− subgroups (5.0% and 5.3%, respectively) and tended to be higher in the GIS+ versus GIS− subgroup (6.0% versus 0.0%, respectively); however, the GIS− group included only six patients.

### 3.4. Disease Response to FL Chemotherapy

**Figure 2:**
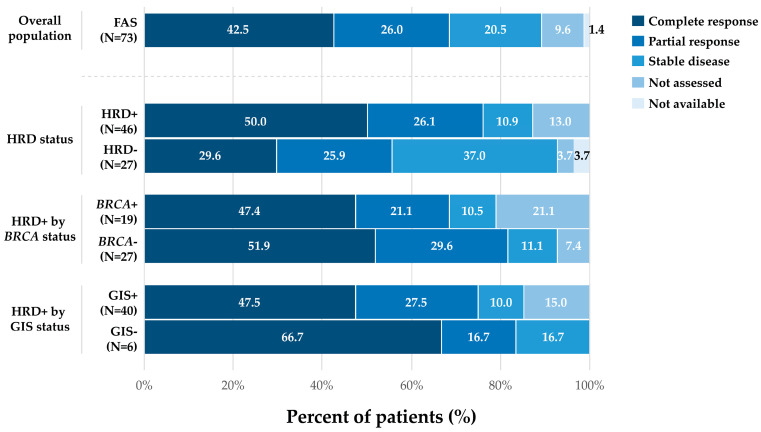
Response to FL chemotherapy-based treatment in patients with advanced OC who started MT treatment and were alive at the time of the second data collection (FAS and by subgroups). Abbreviations: *BRCA*(+/−) = *BReast Cancer* gene (positive/negative); FAS, full analysis set; FL = front-line; GIS(+/−) = genomic instability score (positive/negative); HRD(+/−) = homologous recombination deficiency (positive/negative); MT = maintenance; N = total number of patients; OC = ovarian cancer.

Among 73 patients who started MT and were alive at 6-month follow-up, 42.5% had a complete response to the FL chemotherapy-based regimen, 26.0% had a partial response, and 20.5% had stable disease (Figure 2). The subgroup data are shown in Figure 2.

At 6 months after FL treatment initiation, the mean (SD) serum CA-125 level decreased from 1416.8 (4053.1) to 156.2 (591.3) U/mL in 76 patients with available results. The level of serum CA-125 normalized for most patients, with 78.9% having levels <35 U/mL. The same trend was observed across subgroups (Supplementary Table S1b).

Change from baseline was calculated in 66 patients included in the FAS (paired data). Overall, a median (min, max) reduction of −87.5 (−26,863, 2568) U/mL was observed after excluding the outlier patient (with baseline serum CA-125 level of 81,507 U/mL). Median (min, max) reduction was numerically higher in the HRD− group (−141 [−26,863, 2568] U/mL; *n* = 25) versus the HRD+ group (−85 [−9608, 2484] U/mL; *n* = 40). Among HRD+ patients, median reductions were −96 (−7584, 0) U/mL in the *BRCA*+ subgroup (*n* = 14) and −62 (−9608, 2484) U/mL in the *BRCA*− group (*n* = 26), and −85 (−9608, 2484) U/mL in the GIS+ subgroup (*n* = 34), and −202 (−7584, 0) U/mL in the GIS− subgroup; to note, the number of patients with paired data was small across all groups.

### 3.5. Maintenance (MT) Treatment Patterns

**Figure 3:**
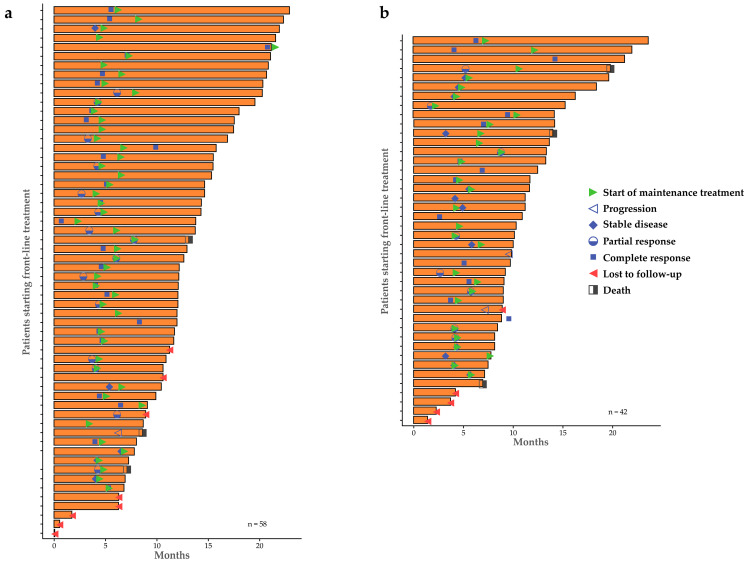
Swimmer plots of time on treatment in individual patients with advanced OC: (**a**) HDR+ group; (**b**) HRD− group (FAS). Abbreviations: FAS, full analysis set; HRD(+/−) = homologous recombination deficiency (positive/negative); *n* = total number of patients; OC = ovarian cancer.

The mean (SD) time from FL treatment initiation to MT treatment start was 5.9 (2.5) months among 77 patients and was similar between the HRD+ and HRD− groups (5.7 [2.7] and 6.1 [2.3] months, respectively) and the *BRCA*+ and *BRCA*− subgroups (5.5 [1.5] and 5.9 [3.3] months, respectively). Time on treatment since initiation of FL treatment to 6-month follow-up by HRD status, including response to FL treatment and start of MT treatment is depicted in Figure 3. The shortest time to MT treatment initiation was observed in the GIS− subgroup (4.9 [0.9] months), while the GIS+ group had a mean of 5.8 [2.8] months; note that the GIS− group included only six patients. 

**Figure 4:**
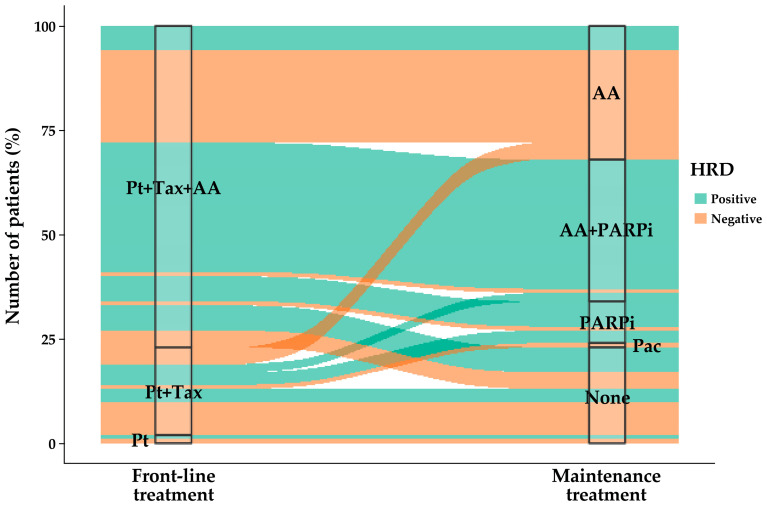
Alluvial plot of the distribution of patients by FL and MT classes used for advanced OC (FAS). Abbreviations: AA = antiangiogenic therapy; FAS, full analysis set; HRD(+/−) = homologous recombination deficiency (positive/negative); MT = maintenance treatment; OC = ovarian cancer; Pac = paclitaxel; PARPi = poly ADP-ribose polymerase inhibitor; Pt = platinum chemotherapy.

Overall, the most frequently used MT treatment was antiangiogenic monotherapy (32.0%) or antiangiogenic therapy + PARP inhibitor (34.0%) (Figure 1b). The most common MT was antiangiogenic monotherapy (19.0%) following FL platinum + taxane doublet chemotherapy, whereas antiangiogenic therapy + PARP inhibitor (41.6%) was most common following FL platinum + taxane doublet chemotherapy + antiangiogenic therapy (Figure 4; Supplementary Table S3a).

Across subgroups, antiangiogenic therapy + PARP inhibitor was the most common MT (range: 50.0–60.5%), except in the HRD− group, where antiangiogenic therapy alone (61.9%) was most common, and in the GIS− subgroup, where MT classes were evenly distributed (33.3%) (Figure 1b). Following FL platinum + taxane doublet chemotherapy + antiangiogenic therapy, the most common MT was antiangiogenic therapy + PARP inhibitor in the HRD+, *BRCA*+, *BRCA*−, and GIS+ subgroups (63.3%, 58.8%, 65.5%, and 65.9%, respectively), whereas antiangiogenic monotherapy was most common in the HRD− group (78.6%). Following FL platinum + taxane doublet chemotherapy, the most common MT was either a PARP inhibitor alone in the HRD+, *BRCA*+, and GIS+ subgroups (37.5%, 100%, 28.6%, respectively) or antiangiogenic therapy + PARP inhibitor in the HRD+, GIS+, and BRCA− subgroups (25.0%, 28.6%, and 40.0%, respectively); antiangiogenic monotherapy (30.8%) was common in the HRD− group (Figure 4, Supplementary Table S3b–d).

Four patients died after MT treatment initiation: two in the HRD− group (received antiangiogenic therapy alone) and two in the HRD+ group (1 *BRCA*+ GIS+ patient received a PARP inhibitor, and one *BRCA*− GIS+ patient received antiangiogenic therapy + PARP inhibitor).

## 4. Discussion

VALIDATE is probably the first real-world evidence study on HRD+ status, clinical characteristics, and the recent treatment landscape (for initial FL and MT) in a cohort of 100 patients with advanced OC from Bulgaria.

HRD represents the inability of tumour cells to effectively repair the DNA double-strand breaks via the HRR pathway [18]. This pathway involves a highly coordinated network of genes and molecular processes that respond to DNA damage to maintain genomic integrity [19]. Given the complexity of the HRR pathway, HRD status can be assessed by either measuring potential causes of HRD (i.e., genetic mutations) or potential effects or consequences of HR loss (i.e., genomic instability) [18,20,21]. The PAOLA-1 trial was one of the first to demonstrate the clinical benefit of combining the PARP inhibitor olaparib with the antiangiogenic agent bevacizumab as first-line MT treatment in patients with newly diagnosed advanced HRD+ OC, regardless of *BRCA* mutation status [22]. In the VALIDATE study population, the HRD positivity rate was 58.0%, being within the ranges reported in pivotal randomized clinical trials investigating PARP inhibitors: PAOLA-1 (48.0%) [22], PRIMA (50.8%) [23], and VELIA (~63%) [24], and a more recent real-world study (65%) in Russian population that utilized the same testing methodology [8]. These comparisons should be interpreted cautiously because the studies differed in HRD assay platform and cut-off definitions (Myriad MyChoice/MyChoice HRD Plus with a cutoff of 42 in PAOLA-1 and PRIMA, MyChoice CDx^®^ with a cutoff of 33 in VELIA, and AmoyDx^®^ HRD Focus Panel with a cutoff of 50 in VALIDATE), as well as in patient selection and treatment context [22,23,24]. Concordance between AmoyDx^®^ HRD Focus Panel and Myriad MyChoiceCDx^®^, which is considered the gold standard but is not available in many regions, was found to be 88.0%, with a positive predictive value of 83.3% and a negative predictive value of 100% for the AmoyDx^®^ test [25]. Moreover, routine-practice estimates may be affected by tumour purity, DNA quality, prior chemotherapy, or local testing practices.

The observed proportion of HRD+ patients (>50% in most studies) supports the relevance of timely genetic testing to inform selection of most adequate treatment approach in advanced OC. HRD positivity has been associated with longer survival outcomes with platinum-based chemotherapy and PARP inhibitors [18,20,26,27]. Despite recognition of HRD as a clinically actionable biomarker, implementation of and access to validated genetic tests depend on multiple factors [28]. This is particularly burdensome in countries where locally validated HRD testing methods are unavailable or not yet reimbursed, including Bulgaria [29,30,31]. Although the lack of government funding did not affect the present study, it highlights a substantial challenge in routine clinical practice in our country. In some instances, support from pharmaceutical companies for biomarkers testing could be a temporary solution for access to advanced diagnostic tools. Integrating biomarkers testing into the public healthcare system remains essential to ensure timely diagnosis and equitable, guideline-concordant care nationwide.

Per protocol, our study enrolled only patients undergoing HRD testing; thus, the true proportion of patients not tested remains unknown. This highlights the need for local studies to evaluate HRD testing rates, particularly following the recent ESGO-ESMO-ESP consensus updates on OC genomic characterization [14]. Currently, the genomic work-up at diagnosis includes testing patients with high-grade non-mucinous tubo-ovarian carcinoma for germline and/or somatic *BRCA1/2* mutations, while HRD testing is recommended in advanced, high-grade non-mucinous tubo-ovarian carcinoma [14]. Implementing genetic testing strategies will help identify high-risk women who could benefit from risk-reduction interventions [11].

In VALIDATE, the study population had a median age of 62 years at the time of advanced OC diagnosis, with younger *BRCA*+ patients (median age of 54 years at diagnosis). Serous histology predominated (91.0% of patients), and the majority of tumours originated in the ovary (95.0%). The patient profile observed in our study is characteristic of patients with advanced OC [8,32,33,34]. However, the surgical approach observed in our study differed from that in a real-world study from the United States (US): the proportion of patients undergoing primary CS was higher (57.0% versus 40.7%, respectively), while the proportion undergoing interval CS was substantially lower (11.0% versus 49.2%, respectively) [33]. Among those who underwent surgery, optimal cytoreduction was achieved in 45.0% of cases, whereas suboptimal cytoreduction was reported in less than 10.0%. Residual disease (R1/R2) was observed in up to 27.0% of cases, a rate reported in the US (28.8%) [33]. Surgical outcome data were missing from the medical records for nearly one-third of patients (28.0%). Our findings highlight the need to improve patient selection for upfront debulking surgery and the overall rate of optimal debulking, as the absence of residual disease is one of the most important prognostic factors in the management of advanced OC [34].

Regarding FL treatment patterns, most patients (77.0%) received platinum + taxane doublet + antiangiogenics, in line with current guideline recommendations [14]. It is important to note that the Bulgarian healthcare system faces substantial challenges due to occasional shortages of critical therapeutic agents, such as carboplatin, which is decisive in OC management. This reflects broader concerns regarding the sustainability and reliability of drug supply chains, which need to be addressed in order to improve treatment accessibility and survival outcomes nationwide. Furthermore, the National Health Insurance Fund does not cover the cost of prescribing tablets, which are primarily used in targeted therapies. In the VALIDATE study, up to one in five patients (23.0%) did not start any MT: in most cases, patients were lost to follow-up (*n* = 13) or died before treatment started (*n* = 2), whereas for the patients still alive (*n* = 8), the reasons for not starting it were not collected. The relatively high number of patients lost to follow-up can be attributed to their mobility in search of improved healthcare, as not all hospitals in Bulgaria have the necessary specialized services or expertise for comprehensive OC management, especially in complex cases involving uncommon tumour types and locations. In addition, the absence of a central electronic health record system accessible across all hospitals further exacerbates patient follow-up challenges. These shortages highlight the need for improved communication, functional networking and interoperability across the country. Optimization of national anti-cancer management strategies and implementation of evidence-based policies are imperative to creating a sustainable oncology ecosystem.

Among 29 HRD− patients who started MT, most (89.6%) received antiangiogenic monotherapy, and the remaining ones received either a combination of antiangiogenic therapy and PARP inhibitor, PARP inhibitor alone, or paclitaxel (3.4% each). Among 48 HRD+ patients who started MT, 68.8% received a combination of a PARP inhibitor and antiangiogenic therapy, and the remaining ones received either a PARP inhibitor (18.8%) or antiangiogenics (12.5%). Currently, only olaparib is available in Bulgaria as MT among PARP inhibitors (the other agents are not approved or available). The variations in treatment approaches observed in our study may be explained by patient (age, frailty, comorbidities, preference), disease (biology, residual disease), physician (preference, experience), and other contextual factors (healthcare reimbursement policies, drug availability) that typically contribute to the multi-layered oncological decision-making process. Moreover, the PRIMA and ATHENA-MONO trials [23,35] reported the small benefits of PARP inhibitors in HDR− patients, and primarily in recurrent settings [36,37,38]. This could be explained by the promotion of non-HRD anti-tumour activity by PARP inhibitors, from immune activation and macrophage reprogramming to decreased ribosomal biogenesis and protein translation [39,40].

Systematized collection of data across multiple sites conducted in VALIDATE provided recent and reliable real-world evidence about the clinical characteristics of patients newly diagnosed with advanced OC tested for HRD status. Yet, the observational/non-interventional nature of the study and inclusion of a preselected population (only those patients who underwent HRD testing) bring limitations, including selection bias, reduced availability of data, and variability in treatment practices, which affect the generalizability of the results. The limited 6-month timeframe chosen for data collection allowed characterization of initial treatment approaches, i.e., FL and first MT, but did not allow any survival analysis, which reduces the clinical impact of our findings. The sample size was small, including only 100 patients, with subsequent smaller subgroup sizes. Statistical tests in the subgroup analyses were exploratory, and results should be interpreted with caution. Preliminary findings obtained require validation in larger populations, with longer follow-up and a more robust study methodology. The minimal amount of treatment-related data in this study prevents any comparison with other studies that assess treatment effectiveness. Lastly, we noted that around one-third of patients lacked general health and oncological parameters (such as weight, BMI, and CA-125 levels), which may be attributed to inconsistent data collection across study centres. Missing data could also have impacted the interpretation of subgroup results, a finding that needs to be addressed and improved for any future research at local level.

## 5. Conclusions

VALIDATE is among the first real-world studies in Bulgaria to describe the characteristics of patients with newly diagnosed advanced OC with HRD status tested. In this cohort, more than one in two patients had HRD+ status, most received FL treatment with platinum–taxane doublet chemotherapy combined with an antiangiogenic agent, with a more heterogenous first MT approach. These findings provide relevant insights into current clinical practice patterns in advanced OC in Bulgaria, serving as a local reference point for future research, with larger samples.

## Figures and Tables

**Table 1:** Characteristics of patients newly diagnosed with advanced OC (FAS and subgroups).

Characteristics	FAS (*N = 100*)	HRD		HRD+ by *BRCA* Status		HRD+ by GIS Status	
		HRD+ (*N = 58*)	HRD− (*N = 42*)	*BRCA*+ (*N = 20*)	*BRCA*− (*N = 38*)	GIS+ (*N = 52*)	GIS− (*N = 6*)
**Age at OC diagnosis, years**							
Mean (SD)	61.3 (10.8)	61.1 (11.1)	61.6 (10.5)	56.1 (9.9)	63.8 (10.9)	61.8 (11.3)	55.1 (7.2)
Median (min–max)	62 (39–86)	62 (39–86)	63 (39–82)	54 (39–76)	64 (44–86)	63 (39–86)	54 (49–69)
>50 years, *n* (%)	84 (84.0)	48 (82.8)	36 (85.7)	14 (70.0)	34 (89.5)	43 (82.7)	5 (83.3)
**Age at FL treatment initiation, years**							
Mean (SD)	61.5 (10.8)	61.3 (11.1)	61.8 (10.5)	56.2 (10.10)	63.9 (10.9)	62.0 (11.3)	55.2 (7.2)
Median (min–max)	62 (39–86)	62 (39–86)	63 (39–82)	54 (39–76)	64 (44–86)	63 (39–86)	54 (49–69)
>50 years, *n* (%)	85 (85.0)	49 (84.5)	36 (85.7)	15 (75.0)	34 (89.5)	44 (84.6)	5 (83.3)
**BMI, kg/m^2^**							
Pts with available results, *n* (%)	69 (69.0)	38 (65.5)	31 (73.8)	14 (70.0)	24 (63.2)	34 (65.4)	4 (66.7)
Mean (SD)	25.9 (6.0)	26.1 (5.1)	25.6 (7.0)	27.5 (5.7)	25.4 (4.6)	25.6 (4.9)	30.9 (4.3)
Distribution, *n* (%)							
<18.5	4 (5.8)	2 (5.3)	2 (6.5)	1 (7.1)	1 (4.2)	2 (5.9)	0 (0.0)
≥18.5–<25	29 (42.0)	14 (36.8)	15 (48.4)	3 (21.4)	11 (45.8)	14 (41.2)	0 (0.0)
≥25–<30	19 (27.5)	12 (31.6)	7 (22.6)	5 (35.7)	7 (29.2)	11 (32.4)	1 (25.0)
≥30–<35	17 (24.6)	10 (26.3)	7 (22.6)	5 (35.7)	5 (20.8)	7 (20.6)	3 (75.0)
**Comorbidities, *n* (%)**							
CVD	46 (46.0)	25 (43.1)	21 (50.0)	6 (30.0)	19 (50.0)	22 (42.3)	3 (50.0)
Respiratory disease	1 (1.0)	1 (1.7)	0 (0.0)	0 (0.0)	1 (2.6)	1 (1.9)	0 (0.0)
Diabetes mellitus	13 (13.0)	10 (17.2)	3 (7.1)	3 (15.0)	7 (18.4)	9 (17.3)	1 (16.7)
Liver disease	1 (1.0)	0 (0.0)	1 (2.4)	-	-	-	-
Other primary malignancies:	10 (10.0)	9 (15.5)	1 (2.4)	4 (20.0)	5 (13.2)	8 (15.4)	1 (16.7)
BC	9 (9.0)	8 (13.8)	1(2.4)	4 (20.0)	4 (10.5)	7 (13.5)	1 (16.7)
Colon cancer	1 (1.0)	1 (1.7)	0 (0.0)	0 (0.0)	1 (2.6)	1 (1.9)	0 (0.0)
Other comorbidities ^1^	27 (27.0)	19 (32.8)	8 (19.0)	4 (20.0)	15 (39.5)	18 (34.6)	1 (16.7)
None	30 (30.0)	15 (25.9)	15 (35.7)	5 (25.0)	10 (26.3)	13 (25.0)	2 (33.3)
Unknown	6 (6.0)	4 (6.9)	2 (4.8)	1 (5.0)	3 (7.9)	4 (7.7)	0 (0.0)
**Family history of malignancies, *n* (%) ^2^**							
BC	6 (6.0)	3 (5.2)	3 (7.1)	2 (10.0)	1 (2.6)	2 (3.8)	1 (16.7)
OC	4 (4.0)	2 (3.4)	2 (4.8)	1 (5.0)	1 (2.6)	1 (1.9)	1 (16.7)
Other malignancies ^3^	9 (9.0)	6 (10.3)	3 (7.1)	4 (20.0)	2 (5.3)	6 (11.5)	0 (0.0)
No	68 (68.0)	38 (65.5)	30 (71.4)	10 (50.0)	28 (73.7)	35 (67.3)	3 (50.0)
Unknown	14 (14.0)	10 (17.2)	4 (9.5)	4 (20.0)	6 (15.8)	8 (15.4)	2 (33.3)
**Primary tumour location, *n* (%)**							
Ovary	95 (95.0)	56 (96.6)	39 (92.9)	20 (100)	36 (94.7)	50 (96.2)	6 (100)
Fallopian tube	3 (3.0)	2 (3.4)	1 (2.4)	0 (0.0)	2 (5.3)	2 (3.8)	0 (0.0)
Peritoneum	2 (2.0)	0 (0.0)	2 (4.8)	-	-	-	-
**Tumour histology, *n* (%)**							
Serous tumour	91 (91.0)	53 (91.4)	38 (90.5)	19 (95.0)	34 (89.5)	48 (92.3)	5 (83.3)
Endometroid tumour	3 (3.0)	2 (3.4)	1 (2.4)	0 (0.0)	2 (5.3)	2 (3.8)	0 (0.0)
Mucinous tumour	2 (2.0)	1 (1.7)	1 (2.4)	1 (5.0)	0 (0.0)	0 (0.0)	1 (16.7)
Undifferentiated carcinoma	2 (2.0)	2 (3.4)	0 (0.0)	0 (0.0)	2 (5.3)	2 (3.8)	0 (0.0)
Other histological type ^4^	2 (2.0)	0 (0.0)	2 (4.8)	-	-	-	-
**Disease stage, *n* (%)**							
Stage II	8 (8.0)	6 (10.3)	2 (4.8)	1 (5.0)	5 (13.2)	5 (9.6)	1 (16.7)
Stage III	51 (51.0)	30 (51.7)	21 (50.0)	11 (55.0)	19 (50.0)	26 (50.0)	4 (66.7)
Stage IV	38 (38.0)	20 (34.5)	18 (42.9)	8 (40.0)	12 (31.6)	19 (36.5)	1 (16.7)
Unknown	3 (3.0)	2 (3.4)	1 (2.4)	0 (0.0)	2 (5.3)	2 (3.8)	0 (0.0)
**ECOG PS at diagnosis, *n* (%)**							
0	61 (61.0)	43 (74.1)	18 (42.9)	13 (65.0)	30 (78.9)	39 (75.0)	4 (66.7)
1	37 (37.0)	15 (25.9)	22 (52.4)	7 (35.0)	8 (21.1)	13 (25.0)	2 (33.3)
2	2 (2.0)	0 (0.0)	2 (4.8)	-	-	-	-
**Cytoreductive surgery type, *n* (%)**							
Primary	57 (57.0)	37 (63.8)	20 (47.6)	14 (70.0)	23 (60.5)	33 (63.5)	4 (66.7)
Interval	11 (11.0)	5 (8.6)	6 (14.3)	3 (15.0)	2 (5.3)	4 (7.7)	1 (16.7)
Unknown	3 (3.0)	2 (3.4)	1 (2.4)	0 (0.0)	2 (5.3)	2 (3.8)	0 (0.0)
No surgery	29 (29.0)	14 (24.1)	15 (35.7)	3 (15.0)	11 (28.9)	13 (25.0)	1 (16.7)

^1^ Other comorbidities included more frequently the following categories of diseases: thyroid diseases (*n* = 7), auto-immune diseases (*n* = 3), psychiatric diseases (*n* = 2) and allergies (*n* = 2). ^2^ Multiple responses per patient could be recorded. ^3^ Other malignancies included the following: colorectal cancer (*n* = 1), head and neck cancer (*n* = 1), hepatocellular carcinoma (*n* = 1), leukaemia (*n* = 1), lung cancer (*n* = 1), malignant melanoma (*n* = 1), stomach cancer (*n* = 2), and unknown malignancy (*n* = 1). ^4^ Other histological types included clear cell tumour (*n* = 1) and anaplastic epithelial carcinoma (*n* = 1). Abbreviations: BC = breast cancer; *BRCA*(+/−) = *BReast Cancer* gene (positive/negative); CVD = cardiovascular disease; ECOG PS = Eastern Cooperative Oncology Group Performance Status; FAS, full analysis set; GIS(+/−) = genomic instability score (positive/negative); HRD(+/−) = homologous recombination deficiency (positive/negative); max = maximum; min = minimum; N = total number of patients; *n* (%) = number (percentage) of patients in a given category; OC = ovarian cancer; SD = standard deviation; - = not applicable.

## Data Availability

The datasets collected and analysed during the study are available from the corresponding author upon reasonable request. Data underlying the findings described in this manuscript may be obtained in accordance with AstraZeneca’s data-sharing policy described at https://astrazenecagrouptrials.pharmacm.com/ST/Submission/Disclosure (accessed on 21 April 2026).

## Supplementary Materials

The following supporting information can be downloaded at: https://www.mdpi.com/article/10.3390/medicina62051000/s1, Figure S1: Study design: data collection timepoints; Table S1: Serum CA-125 levels in patients newly diagnosed with advanced OC at FL treatment start (a) and MT treatment (b) (FAS and by subgroups); Table S2: GSS characteristics in patients newly diagnosed with advanced OC (FAS and by subgroups); Table S3: MT classes used for advanced-stage OC by type of FL treatment in the overall FAS population (a) and by HRD status (b), *BRCA* status (c), and GIS (d).

## Abbreviations

The following abbreviations are used in this manuscript:

AA	Antiangiogenic therapy
ADP	Adenosine diphosphate
BC	Breast cancer
BMI	Body mass index
*BRCA*	*BReast CAncer* gene
*BRCA*(+/−)	Presence/absence of pathogenic *BRCA1/2* mutation
CA-125	Cancer antigen 125
CVD	Cardiovascular disease
DNA	Deoxyribonucleic acid
eCRF	Electronic case report form
ECOG PS	Eastern Cooperative Oncology Group performance status
FIGO	International Federation of Gynaecology and Obstetrics
FAS	Full analysis set
FL	Front-line (treatment)
GCP	Good clinical practice
GIS(+/−)	Genomic instability score (positive/negative)
GPP	Good pharmacoepidemiology practice
GSS	Genomic scar score
HR	Homologous recombination
HRD(+/−)	Homologous recombination deficiency (positive/negative)
MT	Maintenance therapy
OC	Ovarian cancer
PARPi	Poly (ADP-ribose) polymerase inhibitor
Pt	Platinum chemotherapy
R	Statistical programming language R
SD	Standard deviation
TNM	Tumour, node, metastasis classification of malignant tumours
US	United States
