# Supplementary material for: Prevalence of Homologous Recombination Deficiency and Treatment Patterns in Patients with Newly Diagnosed Advanced Ovarian Cancer in Bulgaria: A Real-World Cohort Study (VALIDATE)
Source: Medicina (Kaunas). 2026 May 21;62(5):1000. doi: 10.3390/medicina62051000 (PMC13208283; doi:10.3390/medicina62051000)
Supplement: Supplementary file 1 [file medicina-62-01000-s001.zip › medicina-4213290-supplementary.pdf]

Supplementary materials

Figure S1. Study design: data collection timepoints

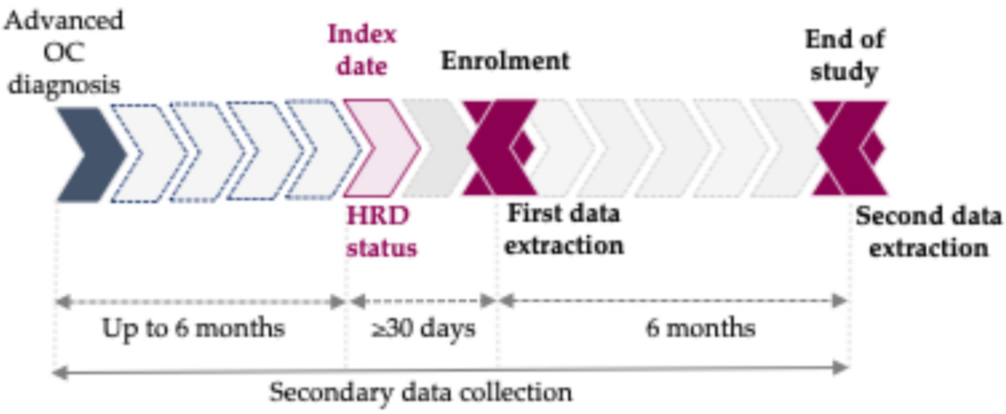

Table S1. Serum CA-125 levels in patients newly diagnosed with advanced OC at FL treatment start (**a**) and MT treatment (**b**) (FAS and by subgroups).

**Serum CA-125 levels, U/mL**	**FAS** *(N=100)*	**HRD**		**HRD+ by *BRCA* status**		**HRD+ by GIS status**	
		**HRD+** *(N=58)*	**HRD–** *(N=42)*	***BRCA+*** *(N=20)*	***BRCA–*** *(N=38)*	**GIS+** *(N=52)*	**GIS–** *(N=6)*
**(a) At FL treatment start (first data extraction)**							
Patients with available results, n (%)	78 (78.0)	45 (77.6)	33 (78.6)	16 (80.0)	29 (76.3)	39 (75.0)	6 (100)
Mean (SD)[1]	1416.8 (4053.1)	870.7 (1913.2)	2145.1 (5756.6)	1062.8 (2072.8)	771.3 (1855.6)	700.1 (1651.5)	1950.7 (3110.8)
Median (min-max)[1]	150 (5-26880)	105 (5-9642)	167 (16-26880)	110 (14-7600)	99 (5-9642)	105 (5-9642)	214 (14-7600)
Patient distribution, n (%)							
0 - <35	19 (24.4)	14 (31.1)	5 (15.2)	4 (25.0)	10 (34.5)	11 (28.2)	3 (50.0)
35 - <500	35 (44.9)	17 (37.8)	18 (54.5)	6 (37.5)	11 (37.9)	16 (41.0)	1 (16.7)
500 - <1500	12 (15.4)	6 (13.3)	6 (18.2)	2 (12.5)	4 (13.8)	6 (15.4)	0 (0.0)
≥1500	12 (15.4)	8 (17.8)	4 (12.1)	4 (25.0)	4 (13.8)	6 (15.4)	2 (33.3)
**(b) At 6 months after FL treatment start (second data extraction)**							
Patients with available results, n (%)	76 (76.0)	45 (77.6)	31 (73.8)	17 (85.0)	28 (73.7)	39 (75.0)	6 (100)
Mean (SD)	156.2 (591.3)	109.7 (549.0)	223.7 (651.2)	50.5 (166.6)	145.7 (686.4)	124.8 (589.2)	11.7 (5.3)
Median (min-max)	12 (1-3647)	10 (1-3647)	17 (3-3181)	10 (3-697)	11 (1-3647)	10 (1-3647)	14 (4-16)

Patient distribution, n (%)							
0 - <35	60 (78.9)	40 (88.9)	20 (64.5)	16 (94.1)	24 (85.7)	34 (87.2)	6 (100)
35 - <500	12 (15.8)	3 (6.7)	9 (29.0)	0 (0.0)	3 (10.7)	3 (7.7)	0 (0.0)
500 - <1500	1 (1.3)	1 (2.2)	0 (0.0)	1 (5.9)	0 (0.0)	1 (2.6)	0 (0.0)
≥1500	3 (3.9)	1 (2.2)	2 (6.5)	0 (0.0)	1 (3.6)	1 (2.6)	0 (0.0)

[1] One patient (HDR+, *BRCA*+, and GIS+) with a baseline CA-125 level of 81530 u/mL was excluded from this analysis because it was considered an outlier.

Abbreviations: *BRCA*(+/–) = *BReast Cancer* gene (positive/negative); FAS, full analysis set; GIS(+/–) = genomic instability score (positive/negative); HRD(+/–) = homologous recombination deficiency (positive/negative); max = maximum; min = minimum; MT = maintenance; n (%) = number (percentage) of patients in a given category; OC = ovarian cancer; SD = standard deviation.

Table S2. GSS characteristics in patients newly diagnosed with advanced OC (FAS and by subgroups).

**GSS characteristics**	**FAS**	**HRD**		**HRD+ by *BRCA* status**		**HRD+ by GIS status**	
	(N=100)	**HRD+ *(N=58)***	**HRD– *(N=42)***	***BRCA*+ *(N=20)***	***BRCA*– *(N=38)***	**GIS+ *(N=52)***	**GIS– *(N=6)***
Mean (SD)	50.4 (42.6)	81.4 (27.9)	7.7 (9.2)	66.5 (38.2)	89.2 (16.2)	89.2 (15.6)	13.4 (13.6)
Median (min-max)	52 (0-100)	96 (3-100)	4 (0-36)	85 (3-100)	98 (50-100)	98 (50-100)	6 (3-36)

Abbreviations: BRCA(+/–) = BReast Cancer gene (positive/negative); FAS, full analysis set; GIS, genomic instability score; GSS = genomic scar score; HRD(+/–) = homologous recombination deficiency (positive/negative); max = maximum; min = minimum; N = total number of patients; OC = ovarian cancer; SD = standard deviation.

Table S3. MT classes used for advanced-stage OC by type of FL treatment in the overall FAS population (**a**) and by HRD status (**b**), *BRCA* status (**c**), and GIS (**d**)

(a)

**MT class, n (%)**	**FL treatment**			
	**Platinum + taxane**	**Platinum + taxane + AA**	**Platinum monotherapy**	**Total**
AA	4 (19.0)	28 (36.4)	0 (0.0)	32 (32.0)
AA + PARP inhibitor	2 (9.5)	32 (41.6)	0 (0.0)	34 (34.0)
PARP inhibitor	3 (14.3)	7 (9.1)	0 (0.0)	10 (10.0)
Paclitaxel	1 (4.8)	0 (0.0)	0 (0.0)	1 (1.0)
No MT treatment started*	11 (52.4)	10 (13)	2 (100)	23 (23.0)
**Total**	**21 (100)**	**77 (100)**	**2 (100)**	**100 (100)**

*Note: 13 patients were lost to follow-up, 2 patients deceased before MT started and among patients alive, 8 patients did not start any MT. AA = antiangiogenic therapy, MT = maintenance treatment; OC = ovarian cancer; PARP = poly-ADP ribose polymerase.

b)

MT class by FL type	HRD+				HRD-				Total			
	**Platinum + taxane**	**Platinum + taxane + AA**	**Platinum mono-therapy**	**Total**	**Platinum + taxane**	**Platinum + taxane + AA**	**Platinum mono-therapy**	**Total**	**Platinum + taxane**	**Platinum + taxane + AA**	**Platinum mono-therapy**	**Total**
AA therapy	0 (0.0)	6 (12.2)	0 (0.0)	***6 (10.3)***	4 (30.8)	22 (78.6)	0 (0.0)	***26 (61.9)***	4 (19.0)	28 (36.4)	0 (0.0)	***32 (32.0)***
AA + PARP inhibitor	2 (25.0)	31 (63.3)	0 (0.0)	***33 (56.9)***	0 (0.0)	1 (3.6)	0 (0.0)	***1 (2.4)***	2 (9.5)	32 (41.6)	0 (0.0)	***34 (34.0)***
PARP in-hibitor	3 (37.5)	6 (12.2)	0 (0.0)	***9 (15.5)***	0 (0.0)	1 (3.6)	0 (0.0%)	***1 (2.4)***	3 (14.3)	7 (9.1)	0 (0.0)	***10 (10.0)***
Paclitaxel	0 (0.0)	-	0 (0.0)	***0 (0)***	1 (7.7)	0 (0.0)	0 (0.0%)	***1 (2.4)***	1 (4.8)	0 (0.0)	0 (0.0)	***1 (1.0)***
No MT treatment started	3 (37.5)	6 (12.2)	1 (100)	***10 (17.2)***	8 (61.5)	4 (14.3)	1 (100%)	***13 (31.0)***	11 (52.4)	10 (13.0)	2 (100)	***23 (23.0)***
**Total**	**8 (100)**	**49 (100)**	**1 (100)**	*58 (100)*	**13 (100)**	**28 (100)**	**1 (100)**	*42 (100)*	**21 (100)**	**77 (100)**	**2 (100)**	*100 (100)*

Abbreviations: AA = antiangiogenic therapy; *BRCA*(+/–) = *BReast Cancer* gene (positive/negative); HRD(+/–) = homologous recombination deficiency (positive/negative); MT = maintenance treatment; PARP = poly-ADP ribose polymerase.

(c)

MT class by FL type	*BRCA+*				*BRCA-*				Total			
	**Platinum + taxane**	**Platinum + taxane + AA**	**Platinum mono-therapy**	*Total*	**Platinum + taxane**	**Platinum + taxane + AA**	**Platinum mono-therapy**	*Total*	**Platinum + taxane**	**Platinum + taxane + AA**	**Platinum mono-therapy**	*Total*
AA therapy	0 (0.0)	3 (17.6)	0 (0.0)	***3 (15.0)***	0 (0.0)	3 (9.4)	0 (0.0)	***3 (7.9)***	0 (0.0)	6 (12.2)	0 (0.0)	***6 (10.3)***
AA + PARP inhibitor	0 (0.0)	10 (58.8)	0 (0.0)	***10 (50.0)***	2 (40.0)	21 (65.6)	0 (0.0)	***23 (60.5)***	2 (25.0)	31 (63.3)	0 (0.0)	***33 (56.9)***
PARP in-hibitor	3 (100)	4 (23.5)	0 (0.0)	***7 (35.0)***	0 (0.0)	2 (6.2)	0 (0.0)	***2 (5.3)***	3 (37.5)	6 (12.2)	0 (0.0)	***9 (15.5)***
No MT treatment started	-	-	-	**-**	3 (60.0)	6 (18.8)	1 (100)	***10 (26.3)***	3 (37.5)	6 (12.2)	1 (100)	***10 (17.2)***
**Total**	**3 (100)**	**17 (100)**	**-**	*20 (100)*	**5 (100)**	**32 (100)**	**1 (100)**	*38 (100)*	**8 (100)**	**49 (100)**	**1 (100)**	*58 (100)*

Abbreviations: AA = antiangiogenic therapy; *BRCA*(+/–) = *BReast Cancer* gene (positive/negative); HRD(+/–) = homologous recombination deficiency (positive/negative); MT = maintenance treatment; PARP = poly-ADP ribose polymerase.

(d)

MT class by FL type	GIS+				GIS-				Total			
	**Platinum + taxane**	**Platinum + taxane + AA**	**Platinum mono-therapy**	**Total**	**Platinum + taxane**	**Platinum + taxane + AA**	**Platinum mono-therapy**	**Total**	**Platinum + taxane**	**Platinum + taxane + AA**	**Platinum mono-therapy**	**Total**
AA therapy	0 (0.0)	4 (9.1)	0 (0.0)	***4 (7.7)***	0 (0.0)	2 (40.0)	-	***2 (33.3)***	0 (0.0)	6 (12.2)	0 (0.0)	***6 (10.3)***
AA + PARP inhibitor	2 (28.6)	29 (65.9)	0 (0.0)	***31 (59.6)***	0 (0.0)	2 (40.0)	-	***2 (33.3)***	2 (25.0)	31 (63.3)	0 (0.0)	***33 (56.9)***
PARP inhibitor	2 (28.6)	5 (11.4)	0 (0.0)	***7 (13.5)***	1 (100)	1 (20.0)	-	***2 (33.3)***	3 (37.5)	6 (12.2)	0 (0.0)	***9 (15.5)***
No MT treatment started	3 (42.9)	6 (13.6)	1 (100)	***10 (19.2)***	0 (0.0)	0 (0.0)	-	-	3 (37.5)	6 (12.2)	1 (100)	***10 (17.2)***
**Total**	**7 (100)**	**44 (100)**	**1 (100)**	***52 (100)***	**1 (100)**	**5 (100)**	**-**	***6 (100)***	**8 (100)**	**49 (100)**	**1 (100%)**	***58 (100)***

Abbreviations: AA = antiangiogenic therapy; *BRCA*(+/–) = *BReast Cancer* gene (positive/negative); HRD(+/–) = homologous recombination deficiency (positive/negative); MT = maintenance treatment; PARP = poly-ADP ribose polymerase.

https://doi.org/10.3390/xxxxx
